# Convergence in Symbiont‐Induced Plant‐Mediated Responses to Herbivory: Cascading Effects for Foraging Parasitoids

**DOI:** 10.1111/ele.70183

**Published:** 2025-08-01

**Authors:** Antonino Cusumano, Serge Urbach, Veronique Jouan, Heiko Vogel, Marcel Dicke, Anne‐Nathalie Volkoff, Erik H. Poelman

**Affiliations:** ^1^ Department of Agricultural, Food and Forest Sciences University of Palermo Palermo Italy; ^2^ Laboratory of Entomology Wageningen University Wageningen the Netherlands; ^3^ IGF, Université de Montpellier, CNRS, INSERM Montpellier France; ^4^ DGIMI Université de Montpellier, INRAE Montpellier France; ^5^ Max Planck Institute for Chemical Ecology Jena Germany

**Keywords:** host discrimination, parasitoid foraging, plant–insect interactions, polydnaviruses, tritrophic interactions

## Abstract

Convergent evolution arises when unrelated species develop similar traits without a shared ancestral origin possessing those characteristics. While typically observed at the organismal level, it can also occur at higher levels of biological organisation. Polydnaviruses represent a striking example of convergent evolution. These viruses, divided into bracoviruses and ichnoviruses, were independently acquired by braconid and ichneumonid parasitoid wasps respectively, to deliver pathogenic genes to caterpillar hosts. Here we show convergent patterns across trophic levels, demonstrating that both bracoviruses and ichnoviruses induce changes in plant‐phenotypic traits that specifically benefit their parasitoid partners, facilitating plant‐mediated host discrimination. This is achieved through an interaction network triggered by changes in the polydnavirus‐infected herbivore (via alteration in regurgitant composition) which eventually affected parasitoids' foraging decisions. Our findings unveil a novel ecological benefit that polydnaviruses offer to their parasitoid partners through intricate, plant‐mediated effects, providing evidence of convergence in symbiont‐induced responses in terrestrial trophic systems.

## Graphical abstract

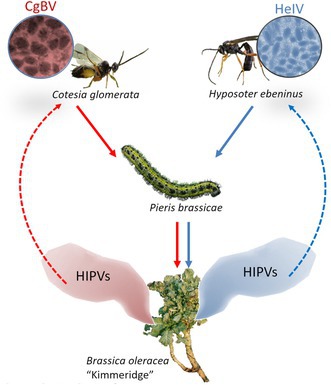

## 1 Introduction

Convergent evolution can be defined as a biological phenomenon where unrelated species develop analogous characteristics or functions, even though they do not share a common evolutionary ancestor that possessed those traits (Sadava et al. 2011). Although convergent evolution can be due to stochasticity or contingency (Vermeij 2006), it commonly occurs when organisms face the same selective pressures or developmental constraints in their environments, leading to the selection of shared traits or adaptations to enhance survival and reproduction (Freeman et al. 2014).

A remarkable case of convergent evolution is represented by polydnaviruses (PDVs), a group of unique insect viruses which have established obligate mutualistic associations with parasitoid wasps more than 100 million years ago (Bezier et al. 2009). This process occurred independently for braconid wasps which acquired PDVs called bracoviruses (BVs), and ichneumonid wasps which acquired ichnoviruses (IVs) (Drezen et al. 2017, 2022). While bracoviruses derive from an ancestral nudivirus, the ancient virus from which ichnoviruses originated has yet to be discovered (Volkoff et al. 2010). PDVs allow parasitoids to overcome the immune barriers of their hosts, usually a caterpillar, in which the immature stages of the parasitic wasp must develop (Chevignon et al. 2015; Doremus et al. 2014; Strand and Burke 2013). Injection of viral particles along with the parasitoid eggs disrupts the host's capacity to mount an encapsulation response towards the eggs, and different genes are used by the two groups of PDVs to achieve this common goal (Pennacchio and Strand 2006; Strand 2012). In their turn, both bracoviruses and ichnoviruses benefit from the symbiotic associations with the parasitoids as viral replication only occurs in the calyx region of the wasp's ovary (Burke and Strand 2014; Strand and Burke 2013).

In recent years, an increasing body of evidence has shown that PDVs do not only manipulate the caterpillar phenotype, as these viral particles can affect the food plant of the infected herbivore and even other plant‐associated insects (Cusumano et al. 2021; Tan et al. 2018; Zhu et al. 2018). These findings have added an extra layer of complexity to the functional characterisation of PDVs. Even if parasitoid‐associated viral particles do not come in contact with plant tissues, they can still profoundly affect the plant phenotype by: (1) regulating the growth rate of the infected herbivores, which in turn alters the amount of feeding damage inflicted on the food plant (Cusumano and Volkoff 2021; Poelman and Cusumano 2022); (2) changing the composition of the herbivore's oral secretions (regurgitant and saliva) which contain elicitors that the plant uses to recognise and mount tailored defence responses against the attacking herbivores (Bonaventure 2012; Rivera‐Vega et al. 2017). These effects have originally been assumed to be triggered by the wasp larvae developing inside the herbivore body (Poelman et al. 2011; Poelman et al. 2012), but it has become clear that in parasitoid species associated with PDVs, these viruses are the main drivers of plant responses to feeding by parasitised herbivores (Dicke et al. 2020). For example, it has been shown that bracoviruses can enhance the fitness of the associated parasitoid species by manipulating plant quality (Tan et al. 2018), although they may impose an ecological cost to the wasp via an interaction network spanning across four trophic levels (Zhu et al. 2018). The discovery that PDVs can affect plant responses to herbivory has questioned to what extent PDV manipulation of the first trophic level can benefit the wasp's fitness and whether viruses act as ‘hidden players’ that drive convergence of biological processes across multiple trophic levels. For example, it remains to be explored whether, in order to enhance wasp fitness, bracoviruses and ichnoviruses induce similar plant‐phenotypic traits, which would suggest patterns of convergence in symbiont‐induced plant‐mediated responses.

**FIGURE 1:**
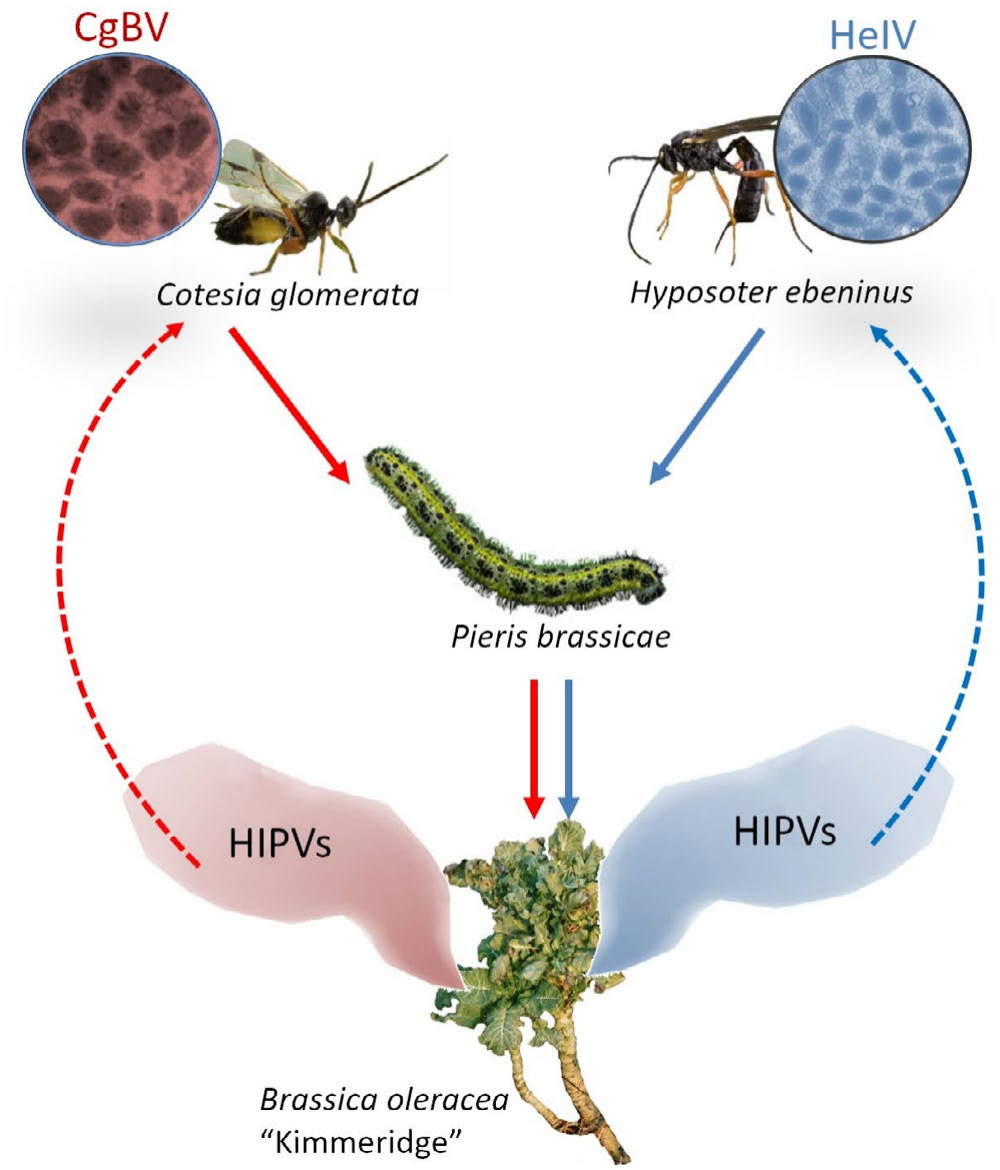
Overview of the *Brassica*‐based tritrophic systems investigated in this study. The parasitoid *Cotesia glomerata* (associated with the bracovirus CgBV) and the parasitoid *Hyposoter ebeninus* (associated with the ichnovirus HeIV) develop in the host *Pieris brassicae* feeding on plants of the wild *Brassica oleracea* population ‘Kimmeridge’. Solid arrows indicate the top‐down effects of the specific polydnaviruses in the food chain (Red = *C. glomerata* bracovirus, Blue = *H. ebeninus* ichnovirus). Dotted lines indicate the ecological effects of the specific polydnavirus on the associated parasitoid species through herbivore‐induced plant volatiles (HIPVs).

Focusing on parasitoid olfactory responses to herbivore‐induced plant volatiles (HIPVs), in this study we explored whether bracoviruses and ichnoviruses induce similar ecological effects in a *Brassica*‐based tritrophic systems (Figure 1). When *Brassica* plants are attacked by herbivores, they release HIPVs which recruit parasitoids of the attacking herbivores (Mattiacci et al. 1995). HIPV emissions are highly informative for foraging parasitoids, as plant volatile composition changes depending on the parasitism status of the attacking herbivore. Due to this specificity of information, it has been shown that the braconid parasitoid *Cotesia glomerata* prefers HIPVs from *Brassica oleracea* plants infested with unparasitised *Pieris brassicae* hosts over HIPVs induced by hosts that have been previously parasitised by conspecific parasitoids (Fatouros et al. 2005). This form of plant‐mediated host discrimination is beneficial for *C. glomerata* as it allows parasitoids to fine‐tune their foraging behaviour towards the discovery of unparasitised host patches, eventually increasing the wasps' reproductive opportunities. Based on previous findings showing that PDVs are responsible for the plant responses to parasitised herbivores, we hypothesise that the bracovirus associated with *C. glomerata* (CgBV) plays a key role in plant‐mediated host discrimination. In *Brassica*‐based food webs, *P. brassicae* caterpillars can also be attacked by the co‐occurring ichneumonid parasitoid *Hyposoter ebeninus* (Poelman et al. 2013). Foraging females of the latter species may also avoid HIPVs induced by caterpillars parasitised by conspecifics and the associated ichnovirus (HeIV) may be responsible for the plant‐mediated effects. Yet, it is not known if plant‐mediated virus‐induced effects facilitate host discrimination in different parasitoid species foraging for shared hosts.

Here, we experimentally manipulated the phenotype of *P. brassicae* caterpillars feeding on *Brassica oleracea* . We isolated the calyx fluid (containing PDV particles) from the parasitoids *C. glomerata* and *H. ebeninus* and injected viral particles into caterpillar hosts that were subsequently feeding on cabbage plants. We discovered that viral symbionts specifically influence the foraging decisions of their associated parasitoid wasps, facilitating plant‐mediated host discrimination (i.e., preference for unparasitised hosts over those parasitised by conspecifics only). We also demonstrate that bracoviruses and ichnoviruses induced distinct signatures in the composition of the regurgitant of infected caterpillars which correlate with parasitoid foraging decisions. Overall, these results elucidate a new ecological benefit that polydnaviruses may provide to their parasitoid partners via complex, plant‐mediated effects and provide evidence of convergence in symbiont‐induced plant‐mediated responses to herbivory.

## 2 Materials and Methods

### 2.1 Plants and Insects

Seeds of the wild *Brassica oleracea* population ‘Kimmeridge’ (Dorset, UK, 50°360 N, 2°070 W) were grown in a greenhouse (22°C ± 3°C, 50%–70% RH and 16:8 h L:D photoperiod). Five‐week‐old plants were used in the experiments. The herbivore *Pieris brassicae* and the parasitoid *Cotesia glomerata* were originally collected from field sites near Wageningen University, the Netherlands, while *Hyposoter ebeninus* was originally collected from cabbage fields near the University of Rennes, France (Harvey et al. 2010). Unparasitised *P. brassicae* caterpillars were reared on cabbage plants ( *B. oleracea var. gemmifera* cv. Cyrus) in glasshouse compartments (22°C ± 2°C, 50%–70% RH and 16:8 h L:D photoperiod). Parasitoids were maintained on *P. brassicae* caterpillars using first and second instar larvae for parasitism. Parasitised caterpillars were reared on cabbage plants in the same conditions as described for unparasitised herbivores.

### 2.2 Isolation of Bracoviruses, Ichnoviruses and Venom

Wasp females were anaesthetised on ice and dissected in phosphate‐buffered saline (PBS) as previously described (Doremus et al. 2013). Calyx fluid (containing the PDV particles) was extracted from the parasitoids *C. glomerata* and *H. ebeninus*. Venom was extracted only for the braconid *C. glomerata* , as venom is reported to synergise the effect of bracoviruses, but not of ichnoviruses (Asgari 2012). In our model system (*H. ebeninus–P. brassicae*), the reduction in growth rate observed in HeIV‐injected caterpillars 48 h post‐injection was similar to that observed in parasitised individuals, indicating that injection of calyx fluid alone is sufficient to reproduce the host developmental changes induced by parasitism (see Supporting Information).

The ovaries and venom apparatus (gland and reservoir) were collected separately and pooled in 250 μL PCR tubes. The volume was adjusted with PBS to reach the desired concentration in wasp equivalents (w.e.) as described by Cusumano et al. (2018, 2021) (e.g., venom apparatus from 30 wasps pooled in 30 μL of PBS for injection of 100 nL containing 0.1 w.e./caterpillar). Venom gland and calyx were disrupted by several passages through a 20 μL micropipette cone. Tubes containing the extracts were centrifuged for 5 min at 2800 G (venom) or for 1 min at 28 G (calyx fluid) and then supernatants containing the venom or calyx extracts were stored on ice until injections were made into caterpillars. It has been shown that purification of the virus by centrifugation has similar effects on caterpillar physiology as other purification techniques such as filtration or using a gradient (Beckage et al. 1994). The presence of PDV particles in calyx extracts was confirmed under an electron microscope Zeiss EM10CR at 80 kV (Cusumano et al. 2018).

### 2.3 Microinjections Into Caterpillars

Second‐instar *P. brassicae* were anaesthetised on ice and injected with PBS solutions with components retrieved from *C. glomerata* (Cg), or *H. ebeninus* (He) using the Eppendorf FemtoJet‐4× injector equipped with glass capillaries (3.5″, Drummond Scientific. No. 3‐000‐203‐G/X). In all experiments, venom, calyx fluid or a mixture of venom and calyx fluid dissolved in 100 nL were injected to prepare the following treatments: (1) *C. glomerata* calyx fluid containing bracovirus particles (CgBV); (2) *C. glomerata* venom (V); (3) *C. glomerata* calyx fluid + venom (CgBV+V); (4) *H. ebeninus* calyx fluid containing ichnovirus particles (HeIV). Additional treatments were used as controls to test whether the microinjection treatment per se affected the interaction of the caterpillars with the food plant: (5) un‐parasitised caterpillars injected with 100 nL of PBS (PBS) (negative controls) and (6) parasitised caterpillars injected with 100 nL of PBS (Cg‐PBS, He‐PBS) (positive controls). Parasitism of *P. brassicae* caterpillars by *C. glomerata* or *H. ebeninus* was performed 2–6 h before injection with PBS as described by Cusumano et al. (2019). After microinjections, caterpillars were allowed to feed on wild *B. oleracea* plants for two days post injection (p.i.) before using them in proteomic analyses. Plants were also induced by injected caterpillars for two days before using them in behavioural assays. This time window was selected because, two days post‐injection (p.i.), the parasitoid progeny of *C. glomerata* and *H. ebeninus* inside parasitised caterpillars is still at the egg stage, eliminating any effects of wasp larval feeding on caterpillar or plant phenotypes.

### 2.4 Collection of Regurgitant, SDS‐PAGE and Quantitative Proteomic Analyses

To investigate whether different injection treatments affect the full protein composition of caterpillar oral secretions, we carried out comparative proteomic investigations of the regurgitant. Regurgitant was collected from caterpillars with a capillary tube (volume = 20 μL) as described in Cusumano et al. (2018) and transferred immediately into a 250 μL Eppendorf tube placed inside a refrigerated block to keep the temperature constantly under 4°C until enough regurgitant was collected. Samples were then centrifuged at 500 rpm for 60 s at 4°C. The supernatant was transferred to new Eppendorf tubes placed inside a refrigerated block and protein concentration was quantified by Bradford spectrophotometric assay (Bradford 1976). Subsequently, samples were stored at −80°C until further use. Each biological replicate consisted of the pooled regurgitant of 15 caterpillars and 3 biological replicates were carried out for each *P. brassicae* (PBS, CgBV+V, HeIV, Cg‐PBS and He‐PBS) treatment. Larvae that had been exposed to oviposition by female wasps were dissected before the regurgitant was pooled to ensure that they were indeed parasitised.

For the purpose of quantitative proteomic analyses, sample preparation was performed using a stacking gel strategy (Muller et al. 2016). Samples (20 μg of protein in 40 μL Laemmli buffer) were loaded onto SDS‐PAGE gels. Then SDS‐PAGE was performed using a short run to achieve minimal separation of the proteins; all bands were excised together and subsequently analysed as a single batch by mass spectrometry. Proteins were subjected to reduction (DTT 1 M, 30 min at 60°C) and alkylation (IAA 0.5 M, 30 min RT). Digestion was performed with trypsin (Gold, Promega, 1 μg/sample, overnight at 37°C). For LC–MS/MS analysis, samples were loaded onto a 25‐cm reversed‐phase column (75 mm inner diameter; Acclaim PepMap 100 C18; Thermo Fisher Scientific) and separated with an UltiMate 3000 RSLC system (Thermo Fisher Scientific) coupled to a QExactive HFX system (Thermo Fisher Scientific) (see Data S1). Spectra were searched against a combination of entries for *P. brassicace* (Proteome Uniprot, http://www.uniprot.org, and IPG from NCBI, May 2024), of the UniProt entries for *B. oleracea* , of the NCBI entries for *Cotesia glomerata* bracovirus (https://www.ncbi.nlm.nih.gov), of the ENA entries for HeIV (accession ERS24611253), of 250 frequently observed contaminants, as well as reversed sequences of all entries. The maximum false discovery rate (FDR) was set to 0.01. LFQ values, obtained with the MaxLFQ normalisation algorithm (implemented in MaxQuant; Cox et al. 2014) were used for comparison between samples (Cox et al. 2014).

### 2.5 Plant Treatments

For the bioassay with *P. brassicae* caterpillars, 5 L2 larvae previously injected as described above were placed on the first fully expanded leaf of a *B. oleracea* Kimmeridge plant. After 48 h of induction, the plants were used in the bioassay to investigate parasitoid odour preference. In bioassays during which caterpillar regurgitant was used, each *B. oleracea* Kimmeridge plant was mechanically damaged on the first fully expanded leaf using a pattern wheel. The wheel was rolled over the leaf surface on each side of the midrib, two lines in parallel (line length 3 cm, distance between each of the lines 0.75 cm) creating a *ca*. 2.25 cm^2^ area with 20 tiny holes (~0.5 mm^2^). A total of 20 μL of regurgitant from differently injected *P. brassicae* caterpillars was applied on these mechanically damaged leaves. To rule out quantitative effects due to plant feeding damage, which differs between unparasitised and parasitised caterpillars, we carried out a set of bioassays with *P. brassicae* caterpillar oral sections inducing mechanically damaged plants. The mechanical damage and regurgitant application were carried out twice (every 24 h) and, after 48 h from the first induction, these plants were used in behavioural bioassays.

### 2.6 Wind Tunnel and Olfactometer Assays

To investigate whether different injection treatments of caterpillars affect the plant‐mediated foraging behaviour of parasitoid females, we carried out olfactory assays using a wind tunnel for *C. glomerata* and a static two‐chamber olfactometer for *H. ebeninus*.

#### 2.6.1 Wind Tunnel

In previous work, it has been shown that *C. glomerata* exhibits plant‐mediated host discrimination to avoid intraspecific competition (Fatouros et al. 2005). We first tested if *C. glomerata* discrimination of HIPVs is still displayed when both parasitised and unparasitised *P. brassicae* caterpillars were injected with saline solution (Cg‐PBS vs. PBS). Then we tested whether this effect is mediated by the *C. glomerata* calyx fluid containing bracoviruses and/or the venom (CgBV vs. PBS; CgBV+V vs. PBS; V vs. PBS). In another set of bioassays, we tested whether plant‐mediated host discrimination is triggered by the caterpillar regurgitant [CgBV+V(reg) vs. PBS(reg)]. Finally, we tested if *C. glomerata* can discriminate between plants induced by caterpillars injected with *H. ebeninus* calyx fluid containing ichnovirus (HeIV) over plants induced by PBS‐injected caterpillars. In all pair‐wise comparisons, *C. glomerata* responses to *B. oleracea* plants were investigated in a wind tunnel following the procedure described by Geervliet et al. (1994), with a wind speed of 0.2 m/s. Plants were placed in the wind tunnel 30 min before the start of the bioassays, then 2‐ to 5‐day‐old, naïve *C. glomerata* female wasps were used which were individually released inside a vial. The vial was placed in the middle of the release cylinder, which was 60 cm downwind from the odour sources. Parasitoid preference for one of the two offered plants was assessed in terms of first choice, which was considered to be made when the wasp landed on a plant. Each treatment combination was replicated with 10 plant pairs and 10 wasps per plant pair.

#### 2.6.2 Static Two‐Chamber Olfactometer Assays

The foraging behaviour of *H. ebeninus* is less investigated in comparison with *C. glomerata*, thus we first tested: (1) whether this species uses HIPVs to locate *P. brassicae* caterpillars feeding on *B. oleracea* plants (PBS) over undamaged plants (UD); (2) whether plant‐mediated intraspecific discrimination occurred (He‐PBS vs. PBS). We then explored the role of *H. ebeninus* calyx fluid which contains ichnoviruses in plant‐mediated discrimination (HeIV vs. PBS) and the role of the caterpillar regurgitant [HeIV(reg) vs. PBS(reg)]. Finally, we investigated if *H. ebeninus* can discriminate between plants induced by caterpillars injected with *C. glomerata* calyx fluid containing bracoviruses alone (CgBV) or in combination with the venom (CgBV+V) over plants induced by PBS‐injected caterpillars. In all pair‐wise comparisons, *H. ebeninus* responses were tested in a static two‐chamber olfactometer as described by Frago et al. (2012). Briefly, this device consists of a glass cylinder, which is divided longitudinally into two identical compartments and closed off with thin mesh. The odour sources placed in each compartment consist of an induced leaf of *B. oleracea* Kimmeridge which was detached from a plant (one leaf/plant) on the morning of the bioassays, placed in glass vials containing tap water and matched with a similar‐sized leaf of the control treatment. Parasitic wasps are released above the mesh in a confined space closed by a glass lid. Parasitoid preference for one of the two *B. oleracea* leaves was assessed in terms of residence time of wasps spent on top of each corresponding chamber. 5 min after placing a source of the stimuli in the olfactometer, a single parasitic wasp was released in the centre of the arena and was given a minute for acclimation, after which its residence time was recorded for an additional period of 5 min. In each bioassay testing a specific leaf pair combination, the response of three female wasps was monitored. To avoid pseudoreplication, the mean response of these 3 wasps was considered a replicate and this procedure was repeated 8 times for each pairwise combination.

### 2.7 Statistical Analyses

Statistical analyses for proteomics data, were performed with Perseus 1.6.15.0 using standard parameters (Tyanova and Cox 2018). After log2 transformation of the LFQ data, only proteins with at least two unique peptides identified in all three replicates of at least one condition were retained. Imputation (‘Replace missing value from normal distribution’ module in Perseus) was used to account for missing values. A principal component analysis (PCA) comparing the proteomic profile of regurgitant from the different *P. brassicae* caterpillar treatments was performed. Differences in terms of protein abundances between *P. brassicae* test groups (either parasitised or virus‐injected) compared to PBS control group were investigated by performing a *t*‐test (FDR = 0.05, s0 = 0.1) with permutation‐based correction.

To investigate plant preference displayed by *C. glomerata* in a wind tunnel, data were analysed using a Generalised Linear Model (GLM) with a binomial distribution and a logit link function (i.e., logistic regression). In particular, to determine under dual‐choice conditions whether there was a significant preference for one of the offered plant treatments, we tested H_0_: logit = 0. A quasi‐binomial distribution was fitted in the model due to overdispersion. To investigate plant preference displayed by *H. ebeninus* in a static two‐chamber olfactometer, a paired *t*‐test was used after verifying that the data did not significantly diverge from normality (Shapiro–Wilk test). We tested the null hypothesis that residence time between the two plant treatments is equal.

## 3 Results

### 3.1 Bracoviruses and Ichnoviruses Induced Specific Changes in the Proteome of Caterpillar Regurgitant

**FIGURE 2:**
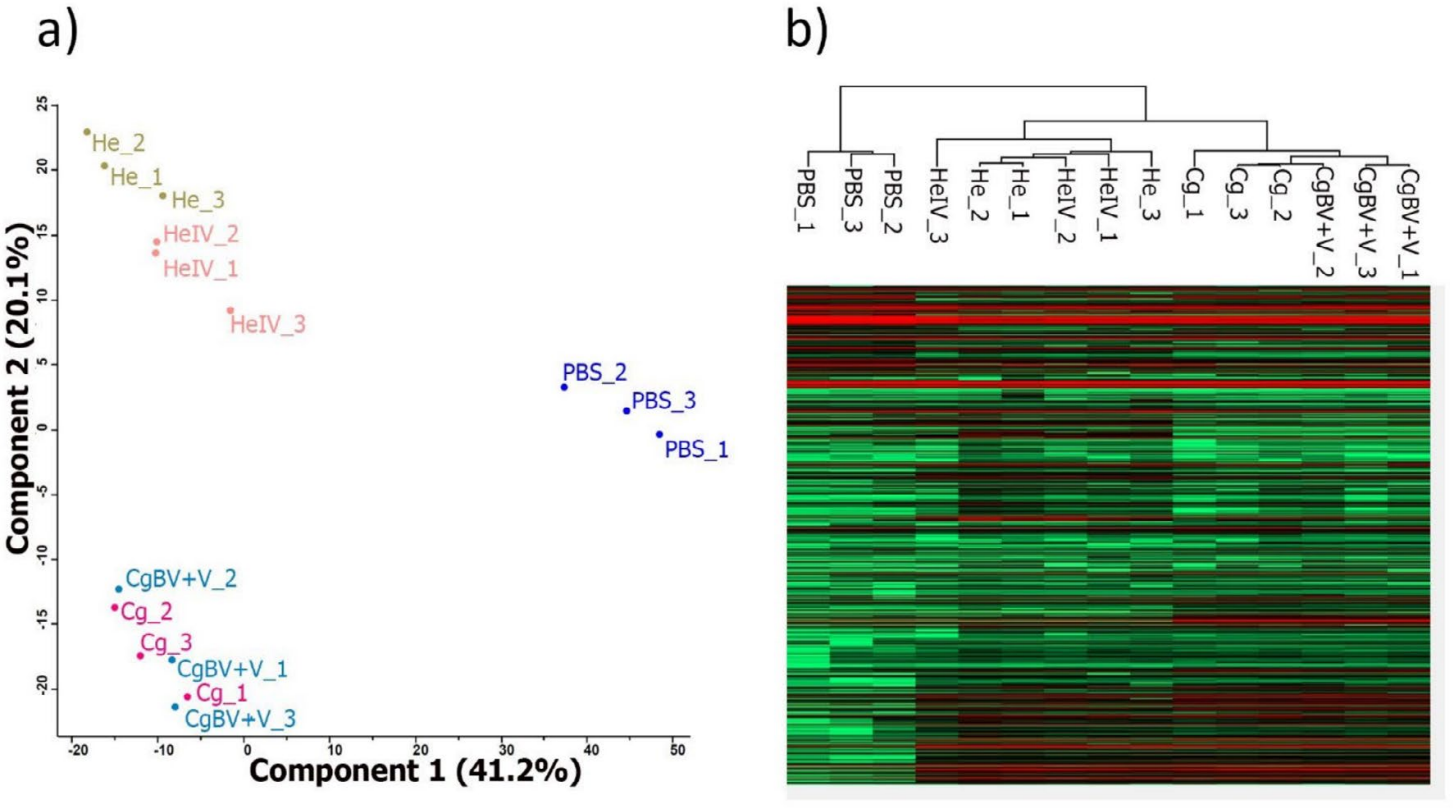
(a) Principal component analysis (PCA) based on proteins (LFQ = log2 protein intensity) detected in the regurgitant of the different *Pieris brassicae* treatments: Blue dots = caterpillars injected only with saline (PBS); dark pink dots= caterpillars parasitised by *Cotesia glomerata* (Cg); light brown dots= caterpillars parasitised by *Hyposoter ebeninus* (He); light blue dots= caterpillars injected with *C. glomerata* bracoviruses and venom (CgBV+V); light orange squares = caterpillars injected with *H. ebeninus* ichnoviruses (HeIV); Total number of proteins identified by mass spectrometry, varied between 614 and 835. (b) Hierarchical clustering based on Pearson correlation (same treatments as above). In the heatmap, colours indicate log2‐transformed LFQ intensities, with green representing lower values (14), black indicating mid‐range values (22), and red representing higher values (30).

Principal component analysis (PCA) of the protein composition of *P. brassicae* regurgitant showed that PBS‐injected caterpillar samples clustered separately from the other treatments, that is, parasitised and PDV‐injected caterpillars (Figure 2a). Interestingly, a large separation occurred between the proteome of caterpillars that were either parasitised by *C. glomerata* (Cg) or injected with its associated bracovirus+venom (CgBV+V) versus those that were either parasitised by *H. ebeninus* (He) or injected with its associated ichnoviruses (HeIV). The first principal component explained 41.2% of the variation, whereas the second component explained 20.1% of the variation. In a similar way, hierarchical clustering separated the treatments, with the control samples (PBS) clustering apart from viral‐injected or parasitised samples (Figure 2b). When plant‐derived proteins present in the regurgitant were left out from the PCA, PBS‐injected caterpillar samples still clustered separately from parasitised and PDV‐injected caterpillars (Figure S1). However, the degree of separation between the proteomes of caterpillars that were either parasitised by *C. glomerata* (Cg) or injected with its associated bracovirus+venom (CgBV+V) and those that were either parasitised by *H. ebeninus* (He) or injected with its associated ichovirus (HeIV) was reduced (Figure S1).

A total of 126 proteins were found in significantly different amounts in the regurgitant of PBS‐injected caterpillars compared to the regurgitant of caterpillars either parasitised by *C. glomerata* (Cg) or injected with its associated bracovirus+venom (CgBV+V). However, most of these proteins corresponded to plant proteins (*n* = 112), and only 14 corresponded to *P. brassicae* proteins (Table S1A). Finding plant (food) material was expected as regurgitant includes part of the insect foregut content. A similar trend was observed when comparing the regurgitant of PBS‐injected caterpillars with that of caterpillars parasitised by *H. ebeninus* (He) or injected with its associated ichnovirus (HeIV): in this case, a total of 122 proteins were found to be significantly different in terms of abundance, including 19 caterpillar proteins and 103 plant proteins (Table S1B). Across both the *C. glomerata* and *H. ebeninus* systems, a total of 21 *P. brassicae* proteins showed significant differences in abundance, with 13 being more abundant and 8 less abundant in parasitised or PDV‐injected samples compared to the PBS control (Table 1). In addition, 12 proteins with altered abundance are shared between the *C. glomerata* and *H. ebeninus* systems, while the others are specific to one of the systems (Table 1, see more details in Appendix S2). The majority of the 21 proteins identified were enzymes, including two β‐glucosidases—well‐known herbivore‐derived elicitors—and other enzymes likely involved in digestion, specifically in carbohydrate, lipid, or protein metabolism (Tables 1, S2). Among plant‐derived proteins, a plant‐apyrase (A0A0D3DXE1) was found to be specifically more abundant in samples associated with *C. glomerata* (Cg, CgBV+V) (Table S1A).

**TABLE 1:** List of the subset of proteins detected in the regurgitant of *Pieris brassicae* caterpillars with increased (positive value) and decreased (negative value) abundance in the presence of PDV virions, that is, treatments with calyx fluid‐injected (CgBV+V = *Cotesia glomerata* bracovirus and venom, HeIV = *Hyposoter ebeninus* ichnovirus) and parasitised caterpillars (Cg = *Cotesia glomerata* , He = *Hyposoter ebeninus*), compared to saline‐injected controls (PBS).

ID	Description	CgBV + V_PBS	Cg_PBS	HeIV_PBS	He_PBS	Predicted signal peptide (SP)	InterPro GO/Biological Process	InterPro GO/Molecular Function
A0A9P0T9E2	Peptidoglycan‐recognition protein LB‐like; WH2 domain‐containing protein	5.046	5.156	4.673	4.932	/	Peptidoglycan catabolism (GO:0009253)	
XP_045533541.1	β‐Glucosidase	4.596	4.468	4.269	4.503	SP	Carbohydrate metabolism (GO:0005975)	Hydrolase (GO:0004553)
A0A9P0TGB4	Lipase member H‐A‐like	1.722	3.752	4.569	5.155	SP	Lipid metabolism (GO:0006629)	Lipase (GO:0016298)
A0A9P0TKP1	Peptidase S1 domain‐containing protein; collagenase‐like	2.381	3.619	2.048	2.466	SP	Proteolysis (GO:0006508)	Endopeptidase (GO:0004252)
A0A9P0TT63	Mucin‐2, partial	0.000	3.045	1.551	3.766	SP	None	None
A0A9P0XE94	Peptidase S1 domain‐containing protein	2.507	2.839	3.219	2.714	SP	Proteolysis (GO:0006508)	Endopeptidase (GO:0004252)
A0A9P0TLS9	Probable phosphoserine aminotransferase	2.194	2.830	1.229	1.181	/	L‐serine biosynthesis (GO:0006564)	Aminotransferase (GO:0004648)
XP_045528590.1	β‐Glucosidase	2.760	2.569	2.861	2.727	SP	Carbohydrate metabolism (GO:0005975)	hydrolase (GO:0004553)
XP_045519396.1	Aminopeptidase N‐like	0.104	2.399	2.386	2.491	SP	Proteolysis (GO:0006508)	Endopeptidase (GO:0004252)
A0A9P0TTW0	Peptidase S1 domain‐containing protein; chymotrypsin‐2‐like	3.654	1.989	1.960	3.262	SP	Proteolysis (GO:0006508)	Endopeptidase (GO:0004252)
A0A9P0TUJ8	Lipocalin/cytosolic fatty‐acid binding domain‐containing protein	2.385	1.964	2.912	3.040	SP	Lipid metabolism (GO:0006629)	Pigment binding (GO:0031409)
A0A9P0TCN3	Aldo‐keto reductase AKR2E4‐like	1.809	1.596	1.951	2.085	SP	None	Oxidoreductase (GO:0016491)
XP_045518659.1	Venom dipeptidyl peptidase 4‐like	0.000	0.453	1.726	2.003	SP	Proteolysis (GO:0006508)	Peptidase (GO:0008236)
A0A9P0SFM2	Haemolymph juvenile hormone binding protein	−1.167	−1.086	−2.299	−2.608	SP		None
A0A9P0T4Z4	Carboxypeptidase B‐like	−1.197	−1.194	−1.564	−2.221	SP	Proteolysis (GO:0006508)	Metallocarboxypeptidase (GO:0004181)
XP_045520632.1	Lipase 3‐like	−1.911	−1.677	−2.042	−2.644	SP	Lipid metabolism (GO:0006629)	Hydrolase (GO:0016788)
A0A9P0TSD2	Defensin	−1.555	−1.991	−2.051	−0.827	SP	None	None
A0A9P0T9C8	Esterase FE4‐like	−2.014	−2.600	−2.384	−0.441	/	None	None
A0A9P0X6G0	Beta‐1,3‐glucan‐binding protein‐like	−2.496	−2.839	−2.126	−2.994	SP	Carbohydrate metabolism (GO:0005975)	Hydrolase (GO:0004553)
A0A9P0TI99	Peptidase S1 domain‐containing protein; trypsin, alkaline B‐like	−2.816	−3.162	−2.492	−3.768	SP	Proteolysis (GO:0006508)	Endopeptidase (GO:0004252)
A0A9P0TD01	Protein D1‐like	−3.404	−4.553	−4.392	−4.722	SP	None	None

*Note:* Proteins shown in this list have a cutoff score in terms of intensity (log2 LFQ differences) > |1|. Statistically significant values (*t*‐test results, based on the geometric means of the three replicates) are colour‐coded: Red indicates proteins with increased abundance in treated samples compared to the PBS control, while blue indicates proteins with decreased abundance. Signal peptides were predicted using the SignalP online tool, and Gene Ontology (GO) terms were assigned using InterPro.

### 3.2 Wind Tunnel

**FIGURE 3:**
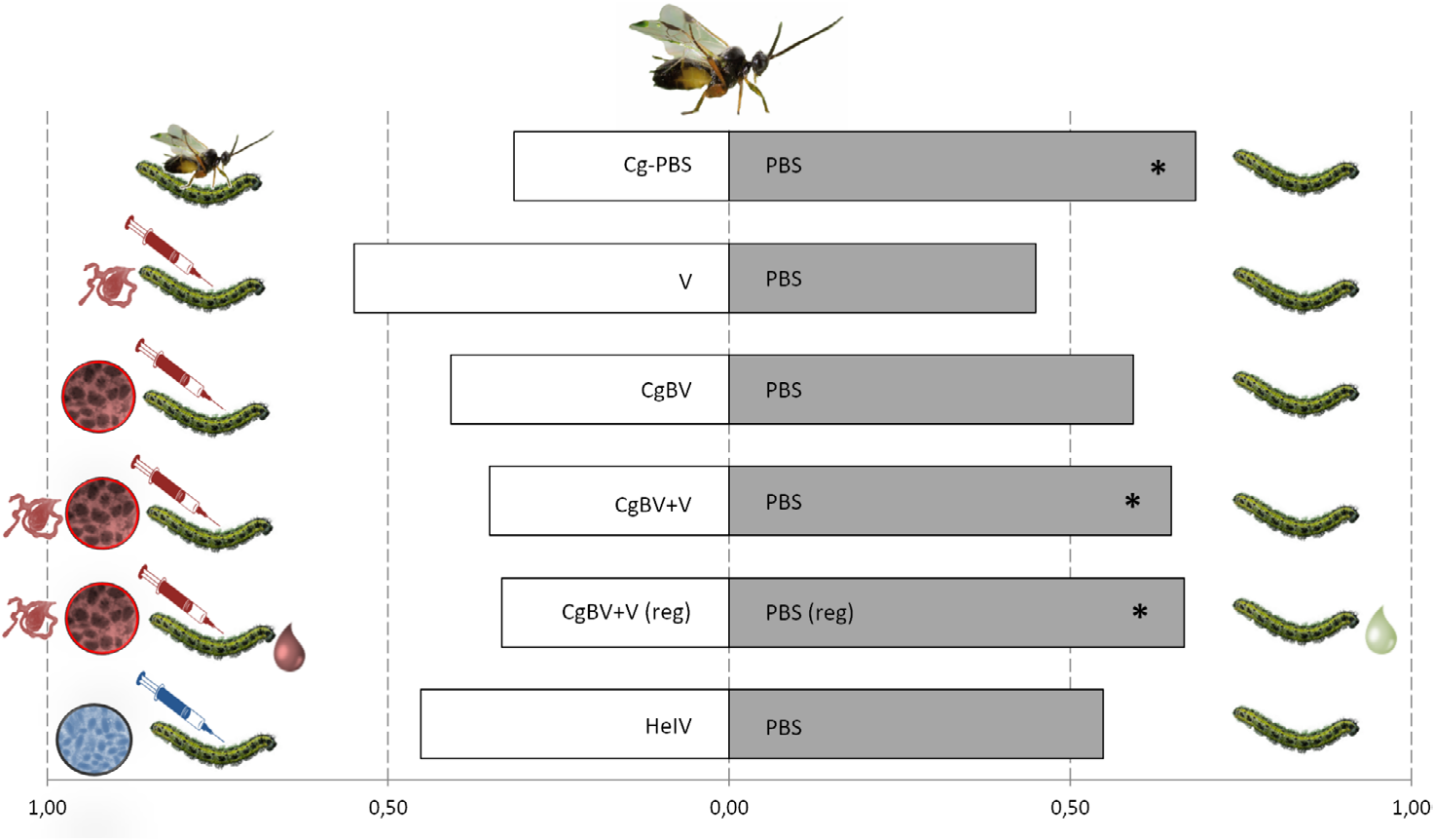
Preference of *Cotesia glomerata* females for herbivore‐induced plant volatiles (HIPVs) in two‐choice wind tunnel tests. *Pieris brassicae* caterpillars feeding on *Brassica oleracea* were subjected to the following treatments: Caterpillars injected only with saline (PBS), caterpillars injected with venom (V), bracoviruses (CgBV) or a combination of both components (CgBV+V) isolated from *C. glomerata* , caterpillars injected with ichnoviruses (HeIV) isolated from *H. ebeninus*, caterpillars parasitized by *C.glomerata* and injected with PBS (Cg‐PBS). CgBV+V(reg) = Plants induced with regurgitant from caterpillars injected with CgBV+V. PBS(reg) = Plants induced with regurgitant from PBS‐injected caterpillars. Asterisks indicate a preference for one of the two pair‐wise plant treatments, which is significantly different from a 50:50 distribution within a choice test (GLM, *p < 0.05).

Females of *C. glomerata* preferred volatiles from *B. oleracea* plants infested by unparasitised *P. brassicae* caterpillars injected with PBS over volatiles from plants infested by parasitised caterpillars injected with PBS (GLM, *t* = 2.61, *n* = 10, *p* = 0.028). Wasps did not discriminate between HIPVs from plants infested by PBS‐injected caterpillars over volatiles from plants infested either by venom‐injected caterpillars (*t* = 1.04, *n* = 10, *p* = 0.324) or caterpillars injected with calyx fluid containing CgBV (*t* = 1.19, *n* = 10, *p* = 0.265) (Figure 3). However, *C. glomerata* 's preference for HIPVs from plants infested by PBS‐injected caterpillars was restored when tested against HIPVs from plants infested by caterpillars injected with both calyx fluid containing CgBV and venom (*t* = 2.85, *n* = 10, *p* = 0.019). Parasitoids preferred plant volatiles induced by regurgitant of PBS‐injected caterpillars over plant volatiles induced by regurgitant of caterpillars injected with a combination of calyx fluid containing CgBV and venom (*t* = 2.96, *n* = 10, *p* = 0.016). Finally, no plant‐mediated discrimination occurred when parasitoids were given a choice between PBS‐injected caterpillars and those injected with *H. ebeninus* calyx fluid containing HeIV (*t* = 0.84, *n* = 10, *p* = 0.426).

### 3.3 Olfactometer Assays

**FIGURE 4:**
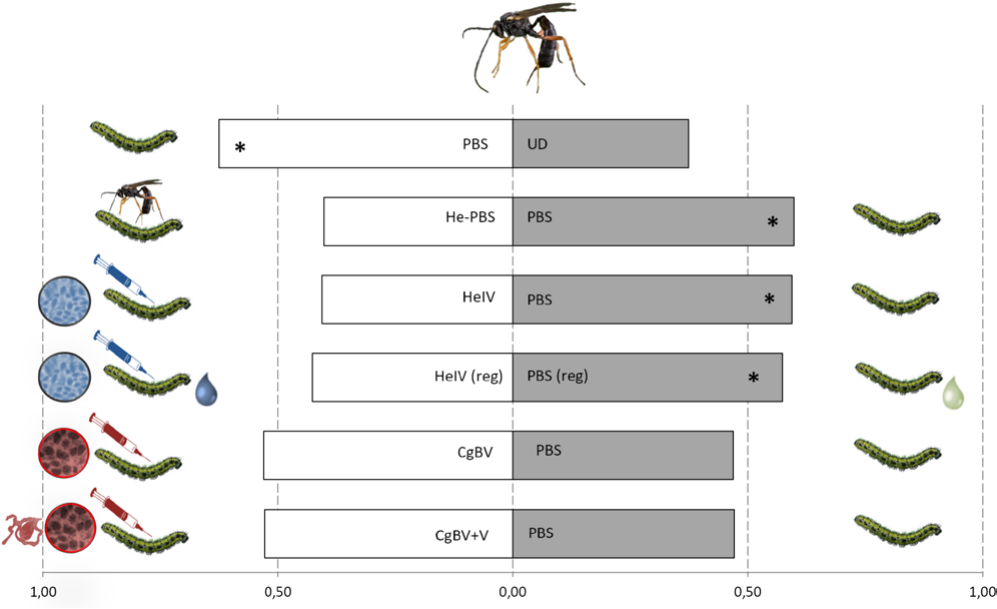
Preference of *Hyposoter ebeninus* females for herbivore‐induced plant volatiles (HIPVs) in two‐choice static olfactometer tests. *Pieris brassicae* caterpillars feeding on *Brassica oleracea* were subjected to the following treatments: Caterpillars injected only with saline solution (PBS), caterpillars injected with ichnoviruses (HeIV) isolated from *H. ebeninus*. Caterpillars injected with bracoviruses alone (CgBV) or in combination with venom (CgBV+V) isolated from *C. glomerata*, caterpillars parasitized by *H. ebeninus* and injected with PBS (He‐PBS) HeIV(reg) = Plants induced with regurgitant from caterpillars injected with HeIV. PBS(reg) = Plants induced with regurgitant from PBS‐injected caterpillars. UD = undamaged plants. Asterisks indicate a preference for one of the two pair‐wise plant treatments, in terms of wasp residence time (Paired *t*‐test, *p < 0.05).

Females of *H. ebeninus* preferred volatiles from *B. oleracea* plants infested by unparasitised *P. brassicae* caterpillars injected with PBS over volatiles of undamaged plants (paired‐*t* test: *t* = 3.60, df = 7, *p* = 0.009) (Figure 4). Wasps preferred HIPVs from plants infested by unparasitised caterpillars injected with PBS over HIPVs from plants infested by parasitised caterpillars injected with PBS (*t* = 4.86, df = 7, *p* = 0.002). Parasitoids preferred plant volatiles induced by regurgitant of PBS‐injected caterpillars over plant volatiles induced by regurgitant of caterpillars injected with *H. ebeninus* calyx fluid containing HeIV (*t* = 2.66, df = 8, *p* = 0.032). Finally, no plant‐mediated discrimination occurred when parasitoids were given a choice between plants infested by PBS‐injected caterpillars and plants infested by caterpillars injected with *C. glomerata* bracovirus either alone (*t* = 1.82, df = 7, *p* = 0.111) or in combination with venom (*t* = 1.3, df = 7, *p* = 0.223).

## 4 Discussion

Bracoviruses and ichnoviruses originated from different ancestors in a remarkable example of convergent evolution where two distinct lineages of parasitoid wasps independently acquired distinct viruses, ultimately for the delivery of viral genes altering the physiology and immunity of caterpillar hosts (Bezier et al. 2009; Volkoff et al. 2010). Here, we expand the knowledge on the functional role of PDVs by showing that both bracoviruses and ichnoviruses induce changes in the regurgitant of infected caterpillars that modify plant‐mediated foraging decisions of parasitoids, eventually facilitating host discrimination. Our results suggest that—by specifically benefitting the associated parasitoid species—PDVs act as ‘hidden players’ mediating the convergence of plant responses across multiple trophic levels.

Intraspecific competition can be intense in insect parasitoids, because hosts represent limited resources. In general, the first parasitoid egg that is laid in a host has a competitive advantage over conspecific eggs, because the hatching larva can start exploiting the limited resources before the other competitors (Cusumano et al. 2016; Harvey et al. 2013). Thus, to optimise foraging strategies and maximise the fitness of their progeny, parasitoid females should be able to discriminate between parasitised and unparasitised hosts. In this perspective, having the ability to exploit HIPVs in order to perceive that caterpillar hosts have been previously attacked by conspecifics seems adaptive for foraging parasitoids. Additionally, parasitoid females would reduce the danger of being bitten or coming in contact with the regurgitant of parasitised caterpillars, which actively defend themselves and can cause serious injuries to parasitoids, a risk particularly common in the case of *C. glomerata* – *P. brassicae* association (Brodeur et al. 1996).

Plant‐mediated intraspecific discrimination in *C. glomerata* has been suggested to be induced by parasitoid larvae developing inside the herbivore's body (Fatouros et al. 2005), but injection of CgBV and venom into caterpillars can mimic the same plant‐phenotypic changes induced by parasitised caterpillars, indicating the key role of the parasitoid associated‐symbiont. Similarly, injection of HeIV triggers plant‐mediated effects on the associated *H. ebeninus* parasitoids. How CgBV and venom act in combination to mimic parasitism‐induced effects from a plant‐insect perspective deserves to be investigated in further studies. CgBV genes might be expressed in the caterpillar's body only in the presence of venom, as observed in the closely related host–parasitoid system *Pieris rapae* – *Cotesia rubecula* , where venom is suggested to assist in the uncoating of viral particles (Zhang et al. 2004). In contrast, in the ichneumonid‐host systems studied so far, venom does not appear to be required for PDV functions (Asgari 2012), and wasps' olfactory responses obtained with HeIV indirectly support this hypothesis. Thus, the functional role of venom appears to be linked to the evolutionary trajectories that followed the independent acquisition of distinct and unrelated viral symbionts by braconids and ichneumonids (Asgari 2012). Altogether our results highlight the strength and specificity of the polydnavirus‐induced signal which cascades across tritrophic levels.

The presence of two co‐occurring parasitoid species ( *C. glomerata* and *H. ebeninus*) that develop in the field in the same herbivore host (Poelman et al. 2013) allowed also to test whether bracoviruses affected ichneumonid parasitoids via HIPVs and *vice versa*. Nonetheless, inter‐specific discrimination was not observed, even in *C. glomerata* , which is an inferior competitor compared to *H. ebeninus* (Poelman et al. 2014). This finding can be explained by considering that the capacity to identify whether a host has been previously parasitised by a different species is uncommon among parasitoids (Van Alphen and Visser 1990). Although the intensity of multiparasitism in the field is not known for the two parasitoid species, intraspecific discrimination is likely a stronger selective pressure, as parasitoids are expected to encounter hosts already parasitised by conspecifics more frequently than by heterospecifics. Consequently, the evolutionary pressure to develop avoidance of hosts parasitised by heterospecific parasitoids may be weak, even for inferior interspecific competitors, although exceptions do exist (Tamo et al. 2006).

The mechanistic effects through which bracoviruses and ichnoviruses alter the foraging behaviour of parasitoid wasps can be either quantitative or qualitative. Especially for PDVs associated with solitary parasitoids such as *H. ebeninus*—which strongly constrain the growth of the infected herbivore and thus reduce the amount of feeding damage inflicted on the plant—quantitative effects may be relevant. Yet, by experimentally controlling the extent of damage inflicted on plant leaves, we determined that both bracoviruses (CgBV) and ichnoviruses (HeIV) affect parasitoid responses to HIPVs primarily through qualitative changes in the composition of the caterpillar regurgitant. This is in agreement with the study by Fatouros et al. (2005) showing that plant induction with regurgitant of parasitised caterpillars attenuated HIPV emission compared to regurgitant of unparasitised caterpillars. Plants can detect feeding by parasitised (or PDV‐injected) caterpillars via changes in the elicitors present in oral secretions and downregulate defence responses accordingly (Cusumano et al. 2018; Tan et al. 2018). Yet, further studies are needed to clarify if a reduction in the emission of HIPVs is driving the parasitoid olfactory responses induced by PDVs observed in this study.

Principal component analysis (PCA) of the protein composition of *P. brassicae* regurgitant showed that PBS‐injected caterpillar samples clustered separately from the other treatments, that is, parasitised and PDV‐injected caterpillars, which is in agreement with the olfactory responses of the parasitoids. Indeed, separation in the PCA occurred between the proteome of caterpillars that were either parasitised by *C. glomerata* (Cg) or injected with its associated bracovirus + venom (BV + V) and those that were either parasitised by *H. ebeninus* (He) or injected with its associated ichnovirus (HeIV). One way to achieve specificity in the composition of the regurgitant could be through the secretion of PDV‐derived peptides, as viral genes have been found to be expressed in the salivary glands of PDV‐infected caterpillars (Bitra et al. 2011; Zhu et al. 2018). However, no PDV‐derived peptides were detected in the regurgitant of parasitised/PDV‐injected caterpillars in this study, although it cannot be excluded that these peptides were below detection threshold given that salivary enzymes are diluted in caterpillar regurgitant. Among insect‐derived proteins, we observed that parasitism by *H. ebeninus* and *C. glomerata* (or injection with their respective PDVs) induced similar changes in the caterpillar proteomics suggesting that only a few peptides trigger plant‐mediated intraspecific discrimination in foraging wasps. Among the peptides that were significantly less abundant compared with PBS‐controls, we found a lipase‐3‐like peptide only in samples associated with *H. ebeninus* (He, HeIV) and it is known that lipases in oral secretions of *Schistocerca gregaria* affect defence responses in *Arabidopsis thaliana* (Schafer et al. 2011). This peptide is a candidate for further studying the mechanistic differences in the olfactory responses of *C. glomerata* and *H. ebeninus*. Interestingly, among the peptides that were significantly more abundant compared with PBS‐controls, we found two β‐glucosidase peptides in *C. glomerata* parasitised caterpillars whereas only one β‐glucosidase was found significantly more abundant in *H. ebeninus* parasitised caterpillars. Considering that β‐glucosidases are well known *P. brassicae* elicitors that induce in cruciferous plants the emission of HIPVs capable of attracting parasitoids including *C. glomerata* (Mattiacci et al. 1995), future studies should test whether variations in the amounts of β‐glucosidases in the regurgitant of differently treated caterpillars may influence the differential olfactory responses of *C. glomerata* and *H. ebeninus*. In our proteomics analyses we found large differences between PBS‐injected controls and the other treatments in the plant‐derived component of caterpillar regurgitant where several peptides were differently abundant, including plant‐β‐glucosidases and plant‐apyrases (whose insect counterparts are known elicitors). In particular, a plant‐apyrase (A0A0D3DXE1) was found to be specifically more abundant in samples associated with *C. glomerata* (Cg, CgBV+V), and might therefore deserve attention in future studies. In addition to suppressing caterpillar immunity, PDVs induced several other effects in the infected caterpillars including developmental arrest (Beckage and Gelman 2004), prevention of metamorphosis (Pruijssers et al. 2009) and reduction of the feeding damage inflicted by caterpillars on plants (Cusumano et al. 2018; Cusumano et al. 2021). It is thus possible to argue that the large variation in plant‐derived proteins observed in our study is due to the effects of parasitism (and PDV infection) on both caterpillar's food intake and digestion. However, to the best of our knowledge we are not aware of any other study that investigated the proteomics of regurgitant in unparasitised and parasitised caterpillars.

Polydnaviruses have traditionally been studied in a bipartite herbivore‐parasitoid context. However, to fully understand the benefits these viruses confer to their parasitoid partners, it is essential to examine such interactions within a multitrophic framework (Dicke et al. 2020; Shikano et al. 2017). By doing so, we discovered patterns of convergence of PDV manipulation across the second‐ and first‐trophic levels, leading to benefits for the associated parasitoids. Thus, our results elucidated that bracoviruses and ichnoviruses, parasitoid‐associated viruses with independent evolutionary histories, can induce fascinating and complex effects on plant‐based trophic systems, broadening our understanding of plant‐insect‐microbe interactions.

## Author Contributions

A.C., M.D., A.‐N.V. and E.H.P. designed the experiments. A.C., S.U., V.J. performed the experiments. A.C. and S.U. analysed the data. A.C., S.U., H.V., M.D., A.‐N.V. and E.H.P. wrote the manuscript.

## Peer Review

The peer review history for this article is available at https://www.webofscience.com/api/gateway/wos/peer‐review/10.1111/ele.70183.

## Supporting information


Data S1.

Table S1.

Table S2.

## Data Availability

The behavioural data supporting the results are archived in ZENODO (https://doi.org/10.5281/zenodo.14684906). The mass spectrometry proteomics data have been deposited to the ProteomeXchange Consortium via the PRIDE repository with the dataset identifier PXD060225 and 10.6019/PXD060225.
