# Supplementary material for: Convergence in Symbiont‐Induced Plant‐Mediated Responses to Herbivory: Cascading Effects for Foraging Parasitoids
Source: Ecol Lett. 2025 Aug 1;28(8):e70183. doi: 10.1111/ele.70183 (PMC12315632; doi:10.1111/ele.70183)
Supplement: Supplementary file 1 — Data S1. [file ELE-28-0-s001.docx]

**To be submitted to:** Ecology Letters

**article type:** Letters

**Convergence in symbiont-induced plant-mediated responses to herbivory: cascading effects for foraging parasitoids**

**running title:** Convergence in symbiont-induced plant-mediated responses

**Authors**

Antonino Cusumano^1,2*^, Serge Urbach^3^, Veronique Jouan^4^, Heiko Vogel^5^, Marcel Dicke^2^, Anne-Nathalie Volkoff^4^, Erik H Poelman^2^

**Supporting Information**

**Figure S1: a)** Principal component analyses (PCA) based on proteins (LFQ = log2 protein intensity) detected in the regurgitant of the different *Pieris brassicae* treatments without the *Brassica oleracea* proteins: red squares = caterpillars injected only with saline (PBS); violet squares = caterpillars parasitized by *Cotesia glomerata* (Cg); light brown squares = caterpillars parasitized by *Hyposoter ebeninus* (He); blue squares = caterpillars injected with *C. glomerata* bracoviruses and venom (CgBV+V); light red squares = caterpillars injected with *H. ebeninus* ichnoviruses (HeIV); **b)** Hierarchical clustering based on Pearson correlation (same treatments as above). ). In the heatmap, colors indicate log2-transformed LFQ intensities, with green representing lower values (14), black indicating mid-range values (22), and red representing higher values (30)


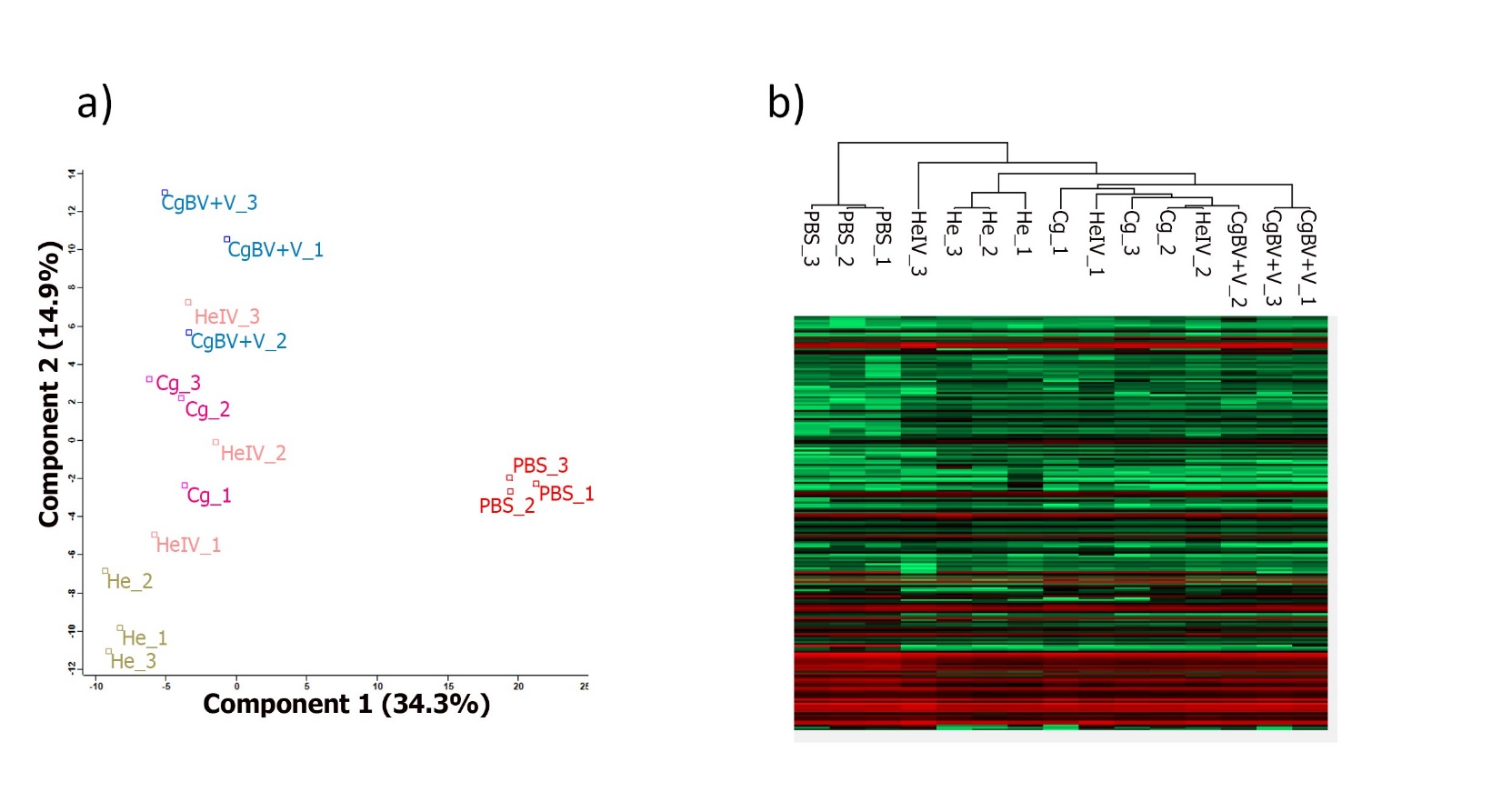


**Appendix S1: Quantitative proteomic analyses**

*Complement to the Materials & Methods section “Collection of regurgitant, SDS-PAGE and quantitative proteomic analyses”.*

Separation of the peptides was performed following a gradient from 2 to 25% buffer B (0.1% AF in 80% ACN) for 100 min at a flow rate of 300 nl/min, then 25–40% for 20 min and finally 40–90% for 3 min. Tandem mass spectrometry analyses were performed in a data-dependent mode. Full scans (375–1,500 m/z) were acquired in the Orbitrap mass analyzer with a resolution of 60,000 at 200 m/z. For MS scans, 3e6 ions were accumulated within a maximum injection time of 60 ms. The 12 most intense ions with charge states ≥ 2 were sequentially isolated (1e5) with a maximum injection time of 45 ms and fragmented by higher-energy collisional dissociation (normalized collision energy of 28) and detected in the Orbitrap analyzer at a resolution of 30,000. Raw spectra were processed with MaxQuant v 2.0.3 using standard parameters with match between runs option.

**Appendix S2: Nature of the proteome of caterpillar regurgitant affected by parasitism or viral infection**

*Complement to the result section “Bracoviruses and ichnoviruses induced specific changes in the proteome of caterpillar regurgitant” and Table 1.*

The proteins whose amount was most decreased in parasitized or PDV-injected regurgitant samples include a D1-like protein of unknown function harboring a phosphatidyl ethanolamine-binding protein (PEBP) domain, a serine-type endopeptidase and a glycosyl hydrolase (see Table 1 and Table S1). On the other side, among the proteins whose amount was most increased in samples from parasitized or PDV-injected caterpillars, there is a peptidoglycan recognition protein, 2 β-glucosidases, a lipase and an endopeptidase. Both under- and overrepresented proteins are shared between the *C. glomerata* and *H. ebeninus* systems (see Table 1). However, analysis of statistically significant peptides indicates that certain *P. brassicae* proteins are specifically affected in only one system: two proteins (A0A9P0TLS9 and XP_045528590.1), both more abundant, are unique to the *C. glomerata* system, while seven proteins are specific to the *H. ebeninus* system - four showing increased abundance (A0A9P0TT63, XP_045519396.1, A0A9P0TCN3, XP_045518659.1) and three showing decreased abundance (A0A9P0SFM2, A0A9P0T4Z4, XP_045520632.1). These differences are further supported by the PCA analysis.

**Table S1.** Proteins showing significant differences in abundance in the regurgitate of *Pieris brassicae*. **Table S1 A:** Comparison of protein abundance after injection of CgBV with venom (CgBV+V) or parasitism by *Cotesia glomerata* (Cg) relative to control injections with PBS (PBS). **Table S1 B:** Comparison of protein abundance after injection of HeIV (HeIV) or parasitism by *Hyposoter ebeninus* (He) relative to control injections with PBS (PBS). Proteins originating from the insect (*P. brassicae*) and the plant (*Brassica oleracea*) are listed separately. For each protein, the following information is provided: the protein identifier (accession number from UniProt [A0A] or NCBI [XP_] databases), protein name (description), number of peptides identified, number of unique peptides, sequence coverage (%), and molecular weight (kDa). Quantitative data includes iBAQ (Intensity-Based Absolute Quantification) values: the number of theoretically detectable tryptic peptides (iBAQ peptides) and the calculated iBAQ intensity. Statistical comparisons are based on LFQ (Label-Free Quantification) values, which offer more accurate estimates of relative protein abundance across samples. For each protein, the log₂(fold change) in LFQ intensity between treatment and control samples (Student’s t-test difference) and the corresponding p-value are reported. Proteins are considered significantly different if the fold change is ≥ 2. Proteins more abundant in treated samples (upregulated) are highlighted in red; those more abundant in control samples (downregulated) are shown in blue.

**Table S2.** List of 21 proteins from *Pieris brassicae* regurgitate that show significantly different abundance in treated samples—either after *Cotesia glomerata* or *Hyposoter ebeninus* exposure—compared to the PBS-injected control (listed in Table 1). For each protein, the following information is provided: the protein ID (accession number from UniProt [A0A] or NCBI [XP_] databases), protein name (description), and the full amino acid sequence. Within each sequence, peptides identified by mass spectrometry are highlighted in red (Peptides column). Each protein sequence was analyzed using [SignalP](https://services.healthtech.dtu.dk/services/SignalP-5.0/) (version 5.0) to predict the presence of a signal peptide, and [InterPro](https://www.ebi.ac.uk/interpro/) was used to assess potential protein function (protein family and GO terms for Biological process and Molecular function are indicated).

**Appendix S3: Growth rate of *Pieris brassicae* caterpillars either parasitized by *Hyposoter ebeninus*, injected with calyx fluid containing HeIV or injected with saline solution**

To investigate whether differently treated *Pieris brassicae* caterpillars displayed phenotypic alterations (i.e. a strong reduction in growth rate) typical of parasitism by solitary species such as *Hyposoter ebeninus*, we quantified their growth rate 48 hours post-injection. Second instar (L2) *P. brassicae* caterpillars were first weighed in a precision scale in order to select caterpillars of very similar weight (range 7.70-8.30 mg). Then they were treated as described in the main text to obtain the following injection treatments: 1) *H. ebeninus* calyx fluid containing ichnovirus particles (HeIV); 2) caterpillars parasitized by *H. ebeninus* and then injected with 100 nL of PBS (He-PBS) (positive controls); 3) un-parasitized caterpillars injected with 100 nL of PBS (PBS) (negative controls). After microinjections, caterpillars were allowed to feed on *B. oleracea* leaves placed inside a petri dish under a climate cabinet (22 ± 2°C, 50–70% RH and 16:8 h L:D photoperiod) for two days. We then reweighted the caterpillars (N= 20 per each treatment) and used ANOVA to test the effect of the fixed factor “caterpillar treatment” on the response variable “weight”. Results indicated that the injection treatment strongly affected the weight of caterpillars (*F* = 64.35, df= 2,57, *P*< 0.001). Caterpillars injected with PBS were significantly larger compared with caterpillars that were infected with ichnoviruses isolated from *Hyposoter ebeninus* (both the treatments “HeIV” and “He-PBS”) (Figure S2). No significant differences in terms of weight were found between parasitized caterpillars and those injected with calyx fluid containing HeIV particles.

**Figure S2.** Weight (mg) by *Pieris brassicae* caterpillars after feeding on *Brassica* leaves for 48 hours. Caterpillars were injected with the following treatments: HeIV = *Hyposoter ebeninus* calyx fluid containing ichnovirus particles; He-PBS = caterpillars parasitized by *H. ebeninus* and injected with PBS; 3) PBS = un-parasitized caterpillars injected with PBS. Different letters above bars indicate significant differences among treatments (ANOVA, P < 0.05)***.***


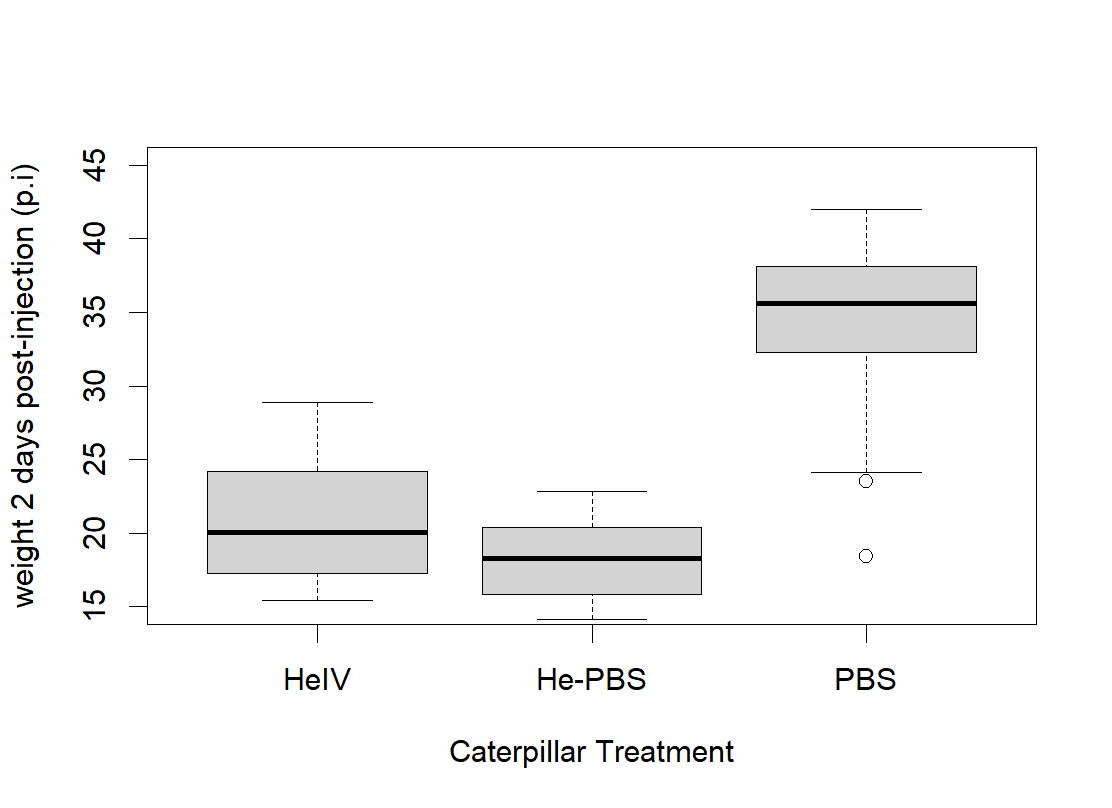


a

b

a
